# Chemotherapy-induced neuropathy in monomethyl Auristatin E treatment: prevention by lithium

**DOI:** 10.1038/s41416-025-03020-6

**Published:** 2025-07-01

**Authors:** Matheus F. Itaborahy, Isadora Z. L. F. Feng, Uri F. Vieira-Machado, Izabela B. da Silveira, Thalita M. Valverde, Guilherme M. J. Costa, Jennifer D. S. Guimarães, Daniela Laet-Souza, Carlos Malamut, M. Alessandra F. Martins, Júlia M. Marques, Julia Rezende-Ribeiro, Vladimir Gorshkov, Frank Kjeldsen, Maria Eduarda S. Favalessa, Izabella F. Acipreste, Thiago Verano-Braga, Hernandes F. Carvalho, André G. Oliveira, Marlon Lemos Dias, Alfredo M. Goes, Barbara E. Ehrlich, M. Fatima Leite

**Affiliations:** 1 Departamento de Fisiologia e Biofísica, Universidade Federal de Minas Gerais, Belo Horizonte, MG Brazil; 2 Departmento de Morfologia, Universidade Federal de Minas Gerais, Belo Horizonte, MG Brazil; 3 Departmento de Bioquímica e Imunologia, Universidade Federal de Minas Gerais, Belo Horizonte, MG Brazil; 4 Centro de Desenvolvimento de Tecnologia Nuclear (CDTN/CNEN), Universidade Federal de Minas Gerais, Belo Horizonte, MG Brazil; 5 Department of Biochemistry and Molecular Biology, University of Southern Denmark, Odense M, Denmark; 6 Departmento de Biologia Estrutural e Funcional, Universidade Estadual de Campinas, Campinas, SP Brazil; 7 Centro de Pesquisa de Medicina de Precisão, Instituto de Biofísica Carlos Chagas, Universidade Federal do Rio de Janeiro, Rio de Janeiro, RJ Brazil; 8 Department of Pharmacology, Yale University School of Medicine, New Haven, CT USA

**Keywords:** Preclinical research, Cognitive control

## Abstract

**Background:**

The increasing number of cancer survivors, thanks to improved cancer treatments, has escalated the prevalence of adverse effects, especially chemotherapy-induced peripheral neuropathy (CIPN) and chemotherapy-induced cognitive impairment (CICI). New drug classes, including antibody-drug conjugates (ADCs), are being developed to target cancer cells and avoid noxious effects. Despite the efforts, ADCs present a high prevalence of neuropathy. A drug often employed in approved ADCs is Monomethyl Auristatin E (MMAE), a microtubule-based agent. The aim of this study was to investigate the sensory and cognitive effects of MMAE in a mouse model and test the potential use of lithium to alleviate MMAE-induced neuropathy.

**Methods:**

We developed a model of MMAE-induced CIPN and CICI and used behavior and sensory tests to analyze these conditions. We also evaluated calcium signaling and protein levels in neuropathic tissues and tumor progression upon treatments with lithium and MMAE.

**Results:**

MMAE administration leads to loss of peripheral sensitivity and cognitive impairment and lithium prevents both central and peripheral neuropathies induced by chemotherapy, without affecting the antitumor activity of MMAE.

**Conclusion:**

This study shows that strategies including lithium pretreatment can prevent both central and peripheral neuropathies induced by chemotherapy to improve quality of life of cancer survivors.

## Introduction

Chemotherapy, while lifesaving, often causes severe adverse events, including chemotherapy-induced peripheral neuropathy (CIPN) and chemotherapy-induced cognitive impairment (CICI). CIPN leads to chronic pain, sensory deficits, and ataxia, while CICI affects memory, learning, focus, and multitasking [1–5]. Up to 75% of cancer patients experience CICI [1, 2]. Treatment for CIPN is only partially effective, and CICI management relies on nonpharmacological approaches. No disease-modifying treatments exist, forcing patients to reduce dosage or duration, compromising efficacy.

Animal models reveal chemotherapy-induced toxicity through altered calcium (Ca²⁺) signaling, apoptosis, inflammation, oxidative stress, and deficits in neurogenesis and neurotransmitter release [6–8]. Despite uncertainties in mechanisms and the lack of therapies, CIPN and CICI are recognized as significant, under-addressed issues.

Taxanes, widely used microtubule-targeting chemotherapeutics, frequently cause neuropathy. New drug classes, including immune modulators and antibody-drug conjugates (ADCs), aim to reduce this toxicity. However, many ADCs use potent microtubule-based agents, still leading to severe neuropathy [2, 9]. As it is anticipated that microtubule-based chemotherapy agents will continue as an important treatment option for cancer patients, the need to prevent CIPN and CICI remains.

Lithium is one of the few agents showing potential for CIPN prevention [10]. Taxanes elevate intracellular Ca²⁺, activating calpain, a Ca²⁺-dependent protease that degrades proteins, disrupts Ca²⁺ signaling, and contributes to neuropathy progression [10, 11]. Lithium prevents this by blocking the initial Ca²⁺ increase, avoiding calpain activation and mitochondrial Ca²⁺ overload, preserving cell function. In mice, lithium did not weaken taxane chemotherapy’s anticancer effects [11].

This study evaluated whether MMAE induces neuropathy and tested lithium as a preventive treatment.

## Materials and methods

### Animals

The experimental procedures done in this study were approved by the Ethics Committee on the Use of Animals of the Federal University of Minas Gerais (CEUA-UFMG) under registration number 201/2023 and in accordance with the relevant guidelines and regulations. The animals were kept in cages inside ventilated racks with a temperature of 22 ± 1 °C and humidity of 40–70%, on a 12/12-h light-dark cycle, and had free access to food and water. All animal experiments were performed during the light cycle. A total of 70 female C57BL/6 mice were purchased from Central Vivarium at UFMG and allowed to habituate to the facility for 7 days. The seven to eight-week-old mice were randomly assigned into 4 groups, with each group separated in its respective cages of 5–6 animals, in accordance with the guidelines provided by UFMG. The mice received intraperitoneal injections of 0.9% saline solution (Farmarin, Brazil) (200 µL), lithium chloride (Sigma-Aldrich, San Luis, Missouri, USA, 12.8 mg/kg in 0.9% saline), Monomethyl Auristatin E (MMAE) (BroadPharm, San Diego, California, USA, 0.12 mg/kg in 1% DMSO in saline), or lithium chloride (12.8 mg/kg in 0.9% saline) co-administered with MMAE (0.12 mg/kg in 1% DMSO in saline). Lithium was administered 1 h before injection of MMAE. The dosage of MMAE is within the dose range capable of causing central and peripheral neuropathies [12, 13]. Drug injections took place every other day over 8 days in a randomized order, to prevent influence from factors such as time of day. Two additional doses of LiCl (6.4 mg/kg and 3.2 mg/kg) were tested together with MMAE to establish a dose-response curve for Von Frey and Capsaicin tests.

### Behavioral tests: open field, elevated plus maze, and novel object recognition

Open-field exploration (OF), elevated plus maze (EPM), and novel object recognition (NOR) tasks were conducted over three consecutive days. The experimental arena was circular (30 cm) and opaque. Mice habituated in the testing room for 1 h before experiments. The OF test analyzed locomotor activity by quantifying movement pattern [10]. The EPM assessed anxiety-like behavior by measuring time spent in open and enclosed arms, as well as entries into open arms [14]. For the NOR task, two 50 mL Falcon tubes were placed in fixed locations to prevent climbing. In the habituation phase, mice explored the arena for 10 min a day before the task. In the familiarization phase, they explored two identical tubes for 10 min. After 20 min, one tube was replaced with a novel object (30 cm PVC tube), and the mouse explored the arena again for 10 min. Video footage of all experiments was analyzed using ANY-maze (7.1), with an investigation zone set to measure interactions. The preference index for the novel object was calculated as 100 × (time spent with novel object / total time with both objects), with the same method applied to the familiar object [15]. The number of animals varied per experiment, adhering to ethical committee guidelines to prevent unnecessary suffering. No stressful tests were conducted before behavioral evaluations.

### Nociception test: capsaicin administration

Capsaicin, an active component of chili peppers, was used to evaluate nociception effects, aiming to characterize peripheral neuropathy caused by the administration of the chemotherapeutic drug. Capsaicin (Sigma-Aldrich, San Luis, Missouri, USA, 1.54 µg in 20 µL in 60% DMSO and 39% PBS) was injected subcutaneously into the dorsal surface of a hind paw. The total time each animal spent licking (total lick time) or shaking the paw was evaluated for 5 min [16]. The experimenters were blinded in relation to experimental groups to avoid confirmation bias.

### Von Frey Test: evaluation of allodynia

Von Frey hairs (0.008 g to 4.0 g, Ugo Basile) were manually applied to assess allodynia in treated mice. Mice habituated for 30–40 min in cylinders on a wire net table before testing. Monofilaments were applied with increasing force using the “ascending stimulus” method [17] until nocifensive responses appeared. Each filament was applied 5 times, with a threshold set at ≥40% positive responses. Blinding prevented confirmation bias. The apparatus was cleaned with 70% ethanol between trials. Group comparisons used the logarithm of force values for analysis [18].

### Euthanasia and tissue collection of C57BL/6

Mice were anesthetized with xylazine (Ceva Santé Animale, São Paulo, Brazil, 10 mg/kg) and ketamine (União Química, São Paulo, Brazil, 80 mg/kg) and then quickly decapitated with scissors. Briefly, the skull was opened, the brain was extracted and washed in 1X phosphate-buffered saline solution. Dorsal Root Ganglia (DRG) were extracted and cleaned after isolation of the spinal column, followed by exposure of the spinal cord [19]. Sciatic nerve was collected from the right leg and cleaned after exposure. Footpads were then removed using a 3 mm punch biopsy.

### FM1-43 staining for afferent sensory fibers of mouse ex vivo DRG

DRGs were surgically removed from control or treated mice and kept in DMEM containing 10% FBS until the experiment. Each DRG was loaded with 4 µM FM™ 1-43 Dye (Invitrogen, Waltham, Massachusetts, USA, Grand Island, NY) for 40 min at 37 °C in a 5% CO_2_ incubator. Then, coverslips containing the tissue were transferred to a chamber on the stage of a confocal microscope. The FM™ 1-43 Dye was used as a fluorescent marker for lipid membranes, to determine whether there were morphological alterations on the DRG axons.

### Coherent Anti-Stokes Raman Spectroscopy (CARS) of sciatic nerve

Sections of the sciatic nerve were stained with the fluoromyelin red staining solution that was prepared by diluting the stock solution 300-fold in PBS. Nerve sections were flooded with the staining solution for 20 min at room temperature, followed by three washes with PBS, each lasting 10 min. Fragments of the sciatic nerve were briefly fixed in 4% paraformaldehyde and maintained in PBS. The fixed material was positioned over a coverslip and immersed in PBS to facilitate imaging with a 40X oil immersion objective. Our configuration employed a Chameleon Discovery NX (Coherent) laser, and a LSM780 (Zeiss) attached to an inverted Microscope with forward and backward non-descanned detectors (PMTs) [20].

### Intracellular Ca^2+^ signaling imaging in mouse ex vivo DRG

DRGs were surgically removed from control or treated mice and kept in DMEM containing 10% FBS until the experiment. Attention was paid to remove surrounding connective tissue around the DRG. Each DRG was loaded with 40 µM Fluo-4/AM (Invitrogen, Grand Island, New York, USA) and 1% Pluronic F-127 (Sigma, St. Louis, Missouri, USA) for 40 min at 37 °C in a 5% CO_2_ incubator. Then, coverslips containing the tissue were transferred to a custom-built perfusion chamber on the stage of a Eclipse Ti microscope (Nikon, RRID:SCR_021242) with an A1R confocal (RRID:SCR_020317) [21]. Intracellular Ca^2+^ signals were monitored upon stimulation with 50 µM carbachol (Sigma, St. Louis, Missouri, USA) as well as 5 mM KCl (Sigma, St. Louis, Missouri, USA), using a 20x objective lens. Ca^2+^ signals were detected and measured by time-lapse setting. Changes in fluorescence upon agonist stimulation (F) were normalized by the baseline fluorescence (F0) and were expressed as (F/F0)*100.

### Real-time PCR of *NCS1*

Total RNA from isolated DRG was extracted using Trizol (Sigma Aldrich) following the user manual. cDNA was synthesized from 1 µg RNA using the High-Capacity cDNA Reverse Transcription Kit (Life Technologies). Real-time PCR was performed with SYBR Green PCR Supermix (Bio-Rad) on a CFX96 Real-Time PCR system (Bio-Rad). Mouse NCS1 primers (Forward: 5’-AAGGCCAGGCAAAAGTGTTC-3’; Reverse: 5’-GCAGTCCTTAATGAAGCCCT-3’) were used. Relative mRNA expression was determined by the comparative Ct method using Bio-Rad software, with beta-actin as the reference gene [22].

### DRG proteomics

Tissue lysis and protein extraction were performed using a buffer with 5% SDC, TEAB, protease/phosphatase inhibitors, and pervanadate (Sigma-Aldrich, Roche). Samples were sonicated, SDC was reduced to 2.5%, and thiols were reduced/alkylated with TCEP and chloroacetamide (Sigma-Aldrich) at 45 °C for 20 min. Proteins were digested with trypsin (1:50) at 37 °C overnight, SDC was precipitated with formic acid, and the supernatant was collected. Peptides were labeled with TMT 16plex, desalted using a C18 column, eluted with ACN/formic acid, and dried in a SpeedVac.

LC-MS/MS analysis was performed using an Orbitrap Eclipse mass spectrometer with HPLC pre-fractionation. The proteomic assay was performed according to Rodrigues-Ribeiro et al. [23].

### Breast cancer xenograft tumor implantation and treatment in Balb/c nude mice

MDA-MB-231 cells tagged with mKate2 (red fluorescent protein) was a donation from the Laboratory of Immunology Transplant - University of São Paulo (USP) and had their identity confirmed using standard analyses of their shape and growth patterns. They were also regularly tested for *Mycoplasma* contamination using 4’,6-diamidino-2-phenylindole staining. Cells were cultured in DMEM with 10% FBS and antibiotic-antimycotic solution (penicillin G sodium, 10 units/mL; streptomycin sulfate, 10 mg/mL; amphotericin B, 0.025 mg/mL) in a humidified 5% CO_2_ atmosphere at 37 °C. For tumor implantation, cells were resuspended in Matrigel (Geltrex™ LDEV-Free Reduced Growth Factor Basement Membrane Matrix) at a density of 3 × 10^6^ cells/50 µL and injected into the dorsal scapular area of female Balb/c nude mice. 2 weeks post-implantation, treatment was initiated as previously described for C57BL/6 mice. During the treatment period, tumor radius was measured and volume was calculated using the semi-ellipsoid formula (4/3πr^3^)/2, normalized to the mouse’s weight [24].

### Immunohistochemistry and fluorescence imaging

Ki-67 was used as a proliferation marker for xenograft tumors in Balb/c nude mice. Paraffin-embedded tissue samples were sectioned and mounted on histological slides as described previously [22]. TUNEL analysis detected apoptotic nuclei using the ApopTag® In Situ Peroxidase Detection Kit (Merck Millipore), following the manufacturer’s protocol. Quantification was based on five representative images (40× objective) from three tissue sections (*n* = 3 each). Formalin-fixed, paraffin-embedded mouse epidermal sections were de-waxed, and antigen retrieval for TuJ-1 (class III β-tubulin, 1:500) was performed using a citrate buffer for 20 min. Sections were treated with 3% hydrogen peroxide for 30 min, blocked with 2.5% horse serum albumin for 1 h, and incubated with TuJ-1 (BioLegend) for 12 h at 4 °C. Detection used a Biotinylated Pan-Specific Universal Antibody (VECTASTAIN® Universal Quick Kit, PK-7800) and DAB (ImmPACT DAB Substrate Kit, SK-4105). Immunostaining was assessed semiquantitatively by optical microscopy. Mouse epidermal samples were from six groups (*n* = 3 per group). Histological quantification was conducted by a blinded investigator, with separate analysis to minimize bias. Skin biopsies are now a standardized tool for diagnosing peripheral neuropathies [25, 26].

### PET-CT imaging of xenograft tumor induced in Balb/c nude mice

A small-animal PET system (LabPET4 Solo, GE Healthcare) was used for imaging and semi-quantitative analysis (SUV). Female Balb/c nude mice were scanned before and two weeks after MDA-MB-231 tumor cell inoculation in the dorsal left scapular area. Mice fasted for at least 4 h, were anesthetized with 2% isoflurane in 100% oxygen, administered 10–14 MBq 18F-FDG, and scanned 60 min post-injection. They remained warmed and anesthetized in a supine position during the 15 min whole-body static acquisition (5 bed positions). Images were reconstructed (128 × 128 × 159 matrix, 0.78 × 0.78 × 0.80 mm) using a 3D-MLEM algorithm with scatter correction [27].

### Statistical analyses

One-way analysis of variance (ANOVA) followed by Bonferroni’s post-hoc tests were used to compare multiple groups. Two-way repeated ANOVA followed by Bonferroni’s post-hoc tests were used to analyze the data from the Novel Object Recognition Test and its training phase. Animals that presented a freezing behavior were excluded from behavior and sensory tests. A *p* < 0.05 was considered statistically significant and the following notations were used in all figures: * for *p* < 0.05, ** for *p* < 0.01, *** for *p* < 0.001, and **** for *p* < 0.0001. For all graphs, error bars shown were standard deviation (SD).

## Results

### MMAE injections do not alter weight, locomotion, or anxiety

**Fig. 1:**
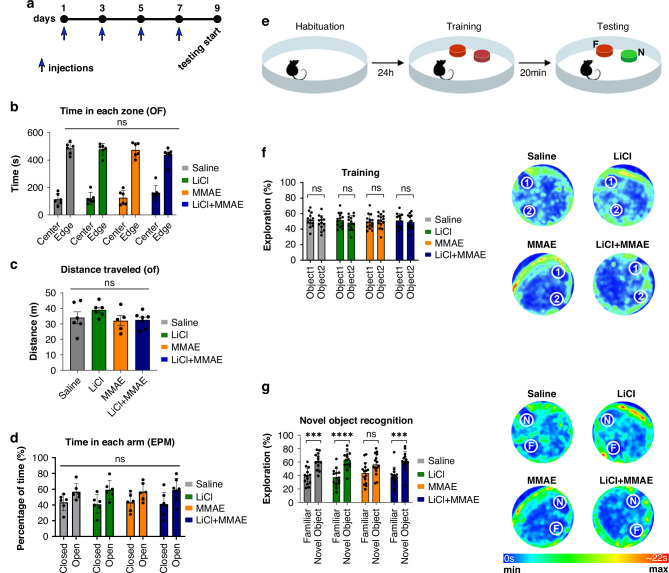
MMAE treatment does not alter the locomotor skills or anxiety-like behavior in mice and Lithium prevents short-term memory loss induced by MMAE in mice. **a** Timeline of the injection protocol for in vivo experiments. **b** The open field test showed no significant difference among the groups in exploration into the center or the edge, indicating no anxiety-like behavior among the groups (*n* = 6 each). **c** The measurement of the distance traveled showed no statistical difference among the groups, suggesting no impact on locomotor skills. **d** The mice had the same tendency to explore in the Elevated Plus Maze, with no significant difference related to anxiety-like behavior among the treatment groups. **e** Visual representation of the Novel Object Recognition Test protocol. **f** Training sessions for the Novel Object Recognition Test showed no statistical difference between the two objects, suggesting no preference for either object. Heat maps from training sessions demonstrating the relative time spent exploring each object (1 = Object 1, 2 = Object 2). **g** Both the saline (*n* = 13) and lithium (*n* = 14) treatment groups were able to identify which object was changed (*p* < 0.001; *p* < 0.0001), whereas the MMAE group (*n* = 15) expressed no significant difference between familiar and novel objects (*p* > 0.05). The LiCl+MMAE group (*n* = 14) showed the ability to differentiate the objects, indicating that lithium pretreatment protects the mice from cognitive impairment induced by MMAE (*p* < 0.001). Heat maps from the Novel Object Recognition Test demonstrating the relative time of exploration for each object (F Familiar Object, N Novel Object).

This study established a mouse model of chemotherapy-induced neuropathy using MMAE, confirmed through nociception and behavioral tests. Mice exhibited normal species-consistent behavior. No significant weight loss differences were observed between treatment and control groups (Supplementary Fig. 1a). Likewise, no significant differences were found in the open field (Fig. 1b, c) or elevated plus maze tests (Fig. 1d), indicating MMAE did not induce anxiety-like behavior or locomotor impairment under the study protocol.

### MMAE administration induces short-term memory impairment that can be prevented with lithium pretreatment

To determine whether MMAE administration induced cognitive impairment, the Novel Object Recognition (NOR) test was employed, targeting short-term memory acquisition. This series of tests also evaluated the effectiveness of lithium co-administration as a strategy to prevent MMAE-induced cognitive impairment. During the training phase, mice showed no preference for either object (*p* > 0.05) (Fig. 1f). During the testing phase, control mice receiving only saline or lithium showed a significant preference for the novel object (*p* > 0.001). In contrast, mice receiving MMAE showed no preference for either object, indicating impaired short-term memory acquisition (*p* > 0.05) (Fig. 1g). When pretreated with lithium, animals in which MMAE was administered maintained the preference for the novel object. These results show that MMAE decreased short-term memory acquisition in mice and that lithium pretreatment prevents this cognitive impairment.

### MMAE administration induces peripheral neuropathy that can be prevented with lithium pretreatment

**Fig. 2:**
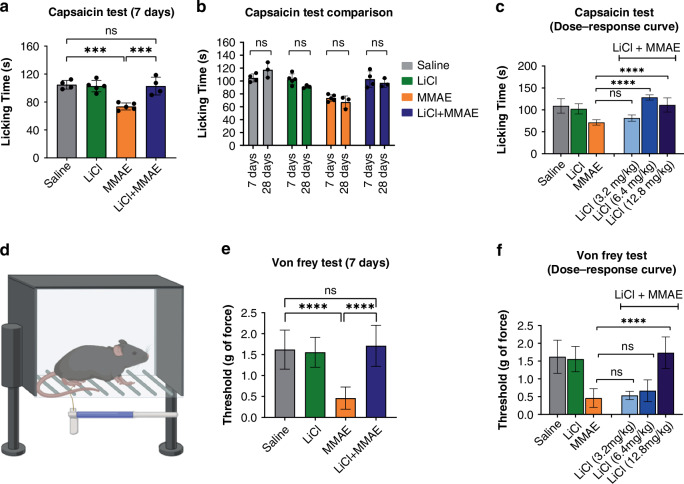
Lithium pretreatment prevented nociceptive impairment and allodynia induced by MMAE. **a** The licking time upon capsaicin injection was significantly different in the MMAE group (*n* = 5) when compared to both the saline (*n* = 4) or lithium (*n* = 5) groups (*p* < 0.001), suggesting loss of peripheral sensitivity to pain. Lithium pretreatment (*n* = 4) prevented nociceptive impairment caused by MMAE-based chemotherapy, with licking time comparable to the control (*p* > 0.05). **b** Comparison of Capsaicin Test results after 7 and 28 days of treatment termination showed no significant difference, indicating that the nociceptive impairment induced by MMAE is sustained up to 28 days and the protection by lithium pretreatment is maintained. **c** A dose-response curve shows that a lower dose of lithium (6.4 mg/Kg) is able to prevent loss of nociception (*p* < 0.0001, *n* = 3 for additional dosages), but not the 3.2 mg/Kg dose of lithium. **d** Graphical representation of the apparatus used for the Von Frey Test. **e** The Von Frey Test showed a significant difference between MMAE group and both saline or lithium groups (*p* < 0.0001), indicating the development of allodynia. Lithium pretreatment was able to prevent the allodynia, showing no statistical difference in the response threshold compared to the control (*p* < 0.0001, *n* = 7 each group). **f** A dose-response curve indicates that lower dosages of lithium are not effective to prevent allodynia in mice (*p* > 0.05, *n* = 3 for additional dosages).

A nociceptive test using capsaicin was performed. Mice exhibited an immediate licking/shaking response post-injection. MMAE-treated mice had reduced licking time (73.6 ± 5.0 s) compared to the saline group (104.7 ± 6.3 s; *p* < 0.001) (Fig. 2a, orange bar). Lithium pretreatment prevented MMAE-induced nociception loss (102.8 ± 12.7 s; *p* < 0.001). This effect persisted 28 days post-treatment (Fig. 2b). Lithium alone did not alter nociception (102.8 ± 8.19 s) (Fig. 2a, green bar). Allodynia, another chemotherapy-induced neuropathy effect, was tested using Von Frey Monofilaments. MMAE reduced the mechanical threshold (3.5 ± 0.3 g) vs. control (4.2 ± 0.1 g; *p* < 0.001) (Fig. 2e), indicating hypersensitivity. Lithium co-administration fully prevented allodynia, with responses similar to control (4.3 ± 0.1 g; *p* > 0.05). Thus, lithium co-administration prevented both nociception loss and allodynia. A lithium dose-response curve in both tests showed lower doses provided reduced CIPN prevention. In the Capsaicin test, 3.2 mg/kg lithium did not significantly improve licking time (*p* > 0.05) vs. MMAE, but 6.4 mg/kg prevented nociception loss (*p* > 0.05 vs. saline) (Fig. 2c). In the Von Frey test, 3.2 mg/kg and 6.4 mg/kg lithium groups showed no significant differences in hypersensitivity vs. MMAE (both *p* > 0.05) (Fig. 2f).

### Lithium prevents peripheral nerve damage induced by MMAE

**Fig. 3:**
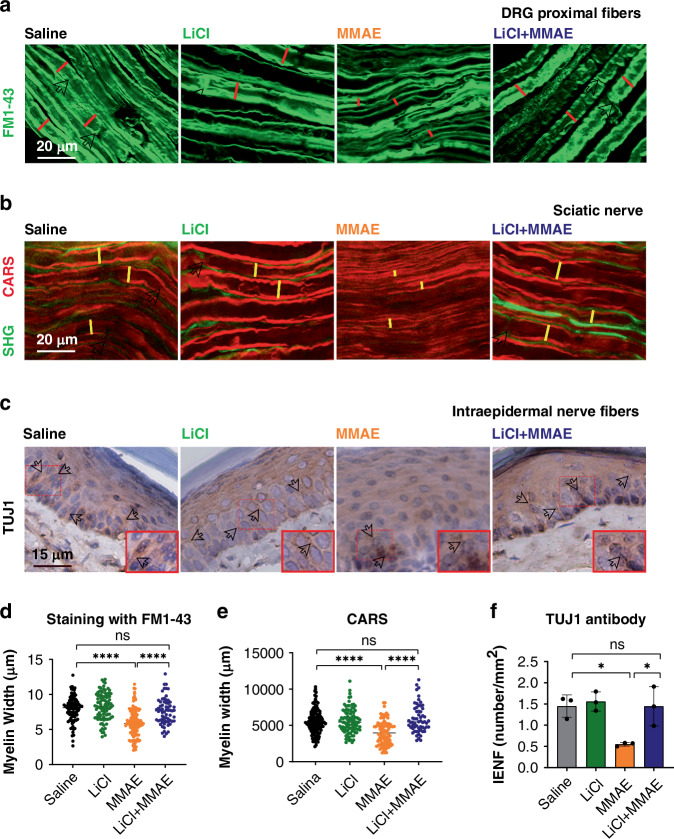
Lithium prevents demyelination and axon shortening in peripheral nerve fibers of animals treated with MMAE. **a** Representative images from ex vivo DRG proximal nerve fibers stained with FM1-43 showed reduction in myelin width in the MMAE group and its prevention in LiCl+MMAE group. White arrows indicate nodes of Ranvier and red traces represent the myelin width, which was measured and quantified in panel 4D. Scale bar 20 μm. **b** Representative images of CARS imaging of sciatic nerve fibers, indicating a similar reduction in myelin width in the group treated with MMAE and its prevention by LiCl coadministration. White arrows indicate nodes of Ranvier and yellow traces represent the myelin width, which was measured and quantified in panel 4E Scale bar 20 μm. **c** Representative images of immunohistochemistry of IENF with TUJ1 antibody, showing a reduction in the number of neuronal terminations in the paw of animals treated with MMAE, which was prevented by lithium treatment. White arrows indicate intraepidermal neuronal terminations. **d** Quantification of the diameter of the ex vivo DRG axons showed reduction in myelin diameter in the MMAE group compared to the saline or lithium groups (*p* < 0.0001, *n* = 3 animals each group). Lithium administration was able to prevent the reduction, with myelin width comparable to the control (*p* > 0.05). **e** Quantification of the myelin diameter in sciatic nerves presented a reduction in the MMAE group compared with saline (*p* < 0.0001, *n* = 3 animals each group), and its prevention by lithium (*p* > 0.05 compared to saline). **f** Quantification of IENF shows a lower number of neuronal terminations in the paw of MMAE treated animals (*p* < 0.05, *n* = 3 animals each group) and its prevention when lithium is used in coadministration (*p* > 0.05 compared to saline).

To assess morphological changes in afferent fibers from the peripheral nervous system, DRG extracted from treated mice were stained with FM1-43 Dye to visualize myelin along axons (Fig. 3a). Myelin thickness was significantly reduced in MMAE-treated mice (5.9 ± 2.0 µm) compared to controls (7.9 ± 1.8 µm; *p* < 0.0001) (Fig. 3d). Lithium co-treatment preserved myelin thickness (7.6 ± 1.9 µm; *p* > 0.05). CARS was employed to visualize lipidic membranes and detect myelin along the DRG axons by utilizing a narrow band-pass filter (660–685 nm) to isolate the CARS signal at 670 nm (Fig. 3b). Quantitative analysis showed significantly thinner myelin in MMAE-treated mice (3.983 ± 0.158 µm) compared to controls (5.519 ± 0.152 µm; *p* < 0.0001) (Fig. 3e). Lithium co-treatment maintained axon widths (5.893 ± 0.247 µm; *p* > 0.05), similar to saline-injected controls. Nerve fiber branching density was assessed in skin biopsies using TuJ-1 staining to visualize small-caliber intra-epidermal nerve fibers (Fig. 3c). The assay was employed to visualize the number and morphology of somatic, small-caliber, intra-epidermal nerve fibers. The number of branches was significantly higher in saline (1.450 ± 0.152 branches/mm²) and lithium (1.558 ± 0.131 branches/mm²) groups compared to MMAE-treated mice (0.551 ± 0.025 branches/mm²; *p* > 0.05) (Fig. 3f). Lithium co-treatment significantly restored branching density (1.446 ± 0.267 branches/mm²; *p* < 0.001 vs. MMAE, *p* > 0.05 vs. saline). These results indicate that MMAE causes substantial peripheral nerve damage, including DRG fiber shrinkage and small fiber neuropathy. Lithium co-treatment effectively prevented degeneration, preserving DRG axon integrity and intra-epidermal nerve morphology.

### MMAE treatment reduces DRG Ca^2+^ signaling amplitude that can be prevented with lithium pretreatment

**Fig. 4:**
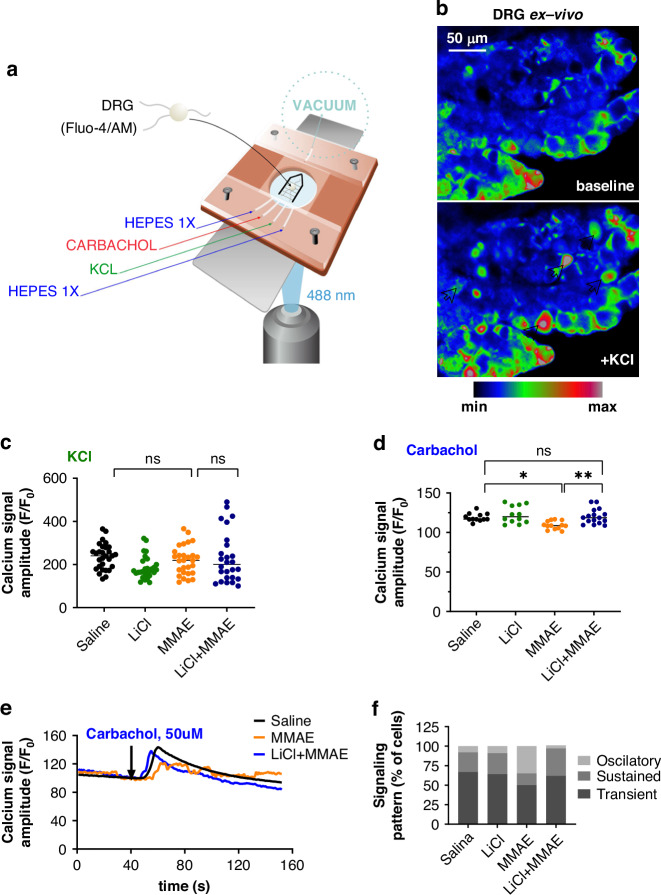
DRG Ca^2+^ signaling amplitude is reduced by MMAE treatment and maintained at normal levels with lithium pretreatment. **a** Schematic representation of the perfusion chamber used for the ex vivo Ca^2+^ signaling experiment. DRGs were removed from mice, loaded with 40 µM Fluo-4/AM and placed in a glass coverslip for perfusion with the indicated stimuli. The DRG was attached to the glass coverslip using a harp shaped metal tool with nylon stripes and intracellular Ca^2+^ signaling was visualized with an excitation wavelength of 488 nm. **b** Representative image of Ca^2+^ signal in ex vivo DRG before and after stimulation with KCl. White arrows indicate cells that responded to KCl stimulus. Scale bar 50 μm. **c** Amplitude of Ca^2+^ signaling in DRG induced by KCl did not differ among the groups, showing that Ca^2+^ influx from the extracellular medium is not impaired by MMAE (*p* > 0.05, *n* = 25–30 cells from 5 mice each group). **d** Amplitude of Ca^2+^ signaling in DRG induced by carbachol was reduced by MMAE, and the reduction was prevented by lithium pretreatment (*p* < 0.05, *n* = 12–16 cells from 5 mice each group). **e** Representative graphic of the time course of Ca^2+^ signaling upon carbachol stimulation in DRG, showing that MMAE not only reduces the amplitude of Ca^2+^ signal, but it also alters the temporal signaling pattern. **f** MMAE treatment increases the number of cells responding with an oscillatory pattern and the LiCl+MMAE pretreatment group primarily responds with transient Ca^2+^ increases.

Previous studies have shown that Ca^2+^, a key regulator of numerous cellular functions, is an important component in the pathway leading to degeneration caused by taxanes [28, 29]. Therefore, measurements of Ca^2+^ transients after stimulation with neuron-specific agonists were performed in ex vivo DRG to assess the effect of MMAE and lithium treatments. We observed that the amplitude of the Ca^2+^ transient upon carbachol stimulation was significantly lower in the MMAE group (109.3 ± 5.2) compared to the control group (119.2 ± 5.2; *p* < 0.01) (Fig. 4d). This result indicates that an alteration of Ca^2+^ signaling occurs with MMAE treatment and is part of the pathway leading to CIPN. Moreover, in the MMAE-treated group a change in the pattern of the Ca^2+^ transient was also observed, with a higher number of cells responding in an oscillatory manner (Fig. 4f). Lithium was able to prevent the changes in the Ca^2+^ signaling pattern as well as the reduction in the Ca^2+^ transient amplitude, which presented values comparable to the control group (121.2 ± 10.4; *p* > 0.05). However, when KCl was used as the stimulus, no difference was observed among the groups (*p* > 0.05) (Fig. 4c). Carbachol acts as an agonist for both muscarinic (G protein-coupled) and nicotinic (ionotropic) receptors, whereas KCl acts only by activation of voltage-operated Ca^2+^ channels [30]. Therefore, a selective alteration in the response to carbachol, but not to KCl, indicates that the reduction of Ca^2+^ signal amplitude comes primarily from alterations in Ca^2+^ release from the endoplasmic reticulum, rather than Ca^2+^ flux across the plasma membrane. These results show that the pathological pathways associated with MMAE treatment involve an impairment of the cellular components that regulate intracellular Ca^2+^ signals. Lithium is also able to maintain the intracellular Ca^2+^ signaling pattern comparable to the control pattern.

### Insights of the mechanism: protein expression in DRG

The role of neuronal calcium sensor-1 (NCS1) in chemotherapy-induced neuropathy has been described for taxane treatment, where its expression decreases soon after administration and remains low post-chemotherapy [10, 11]. To evaluate whether NCS1 is involved in MMAE-induced cognitive impairment, we measured its expression via RT-PCR in treated mouse brains. No significant alterations were found in NCS1 mRNA levels in any group (*p* > 0.05) (Supplementary Fig. 3), suggesting that MMAE-induced dysfunction differs from taxane-induced CIPN [11]. A Live & Dead assay performed in DRG taken from treated mice showed that no differences were observed among groups in terms of cell death (Supplementary Fig. 2), indicating that the pathology of CIPN may not necessarily involve death.

**Fig. 5:**
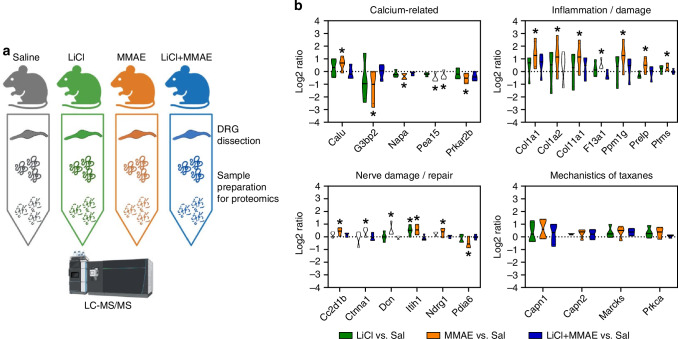
Proteomic evaluation of DRG from treated mice. **a** Schematic representation of the protocol used for the proteomic assay. **b** Mean protein levels in DRG from LiCl, MMAE and LiCl+MMAE groups in comparison to the control divided into functional groups: Ca^2+^-Related proteins, proteins related to Inflammation and Tissue Damage and proteins that are linked to Nerve Damage and Repair (*n* = 5 animals each group). Proteins from the classic pathological mechanism of paclitaxel are also presented, with no significant alterations caused by MMAE or lithium treatments (*p* > 0.05).

A proteomics assay was performed on DRG to identify mechanisms underlying neuronal morphology changes and impaired intracellular Ca²⁺ signaling (Fig. 5, Supplementary Table 1). Analyzing the results, we grouped proteins in 3 functional groups: Ca^2+^-Related proteins, proteins related to Inflammation and Tissue Damage and proteins that are linked to Nerve Damage and Repair.

Calumenin (Calu), a Ca^2+^-binding protein localized in the endoplasmic reticulum, is significantly increased in MMAE-treated DRG, and its up-regulation has been previously shown in injured rat nerves (*p* < 0.05) [31, 32]. Other proteins that are associated with intracellular Ca^2+^ signaling were also found to be altered by MMAE, and mainly down-regulated, such as Ras GTPase-activating protein-binding protein 2 (G3bp2), Alpha-soluble NSF attachment protein (Napa) (N-ethylmaleimide-sensitive factor attachment protein alpha), cAMP-dependent protein kinase type II-beta regulatory subunit (Prkar2b) and Astrocytic phosphoprotein (Pea15) (*p* < 0.05). Previous studies demonstrate that changes in expression and regulation of Pea15 are present in several diseases including cancer, diabetes mellitus, cardiovascular disease and, in particular, neurological disorders. Interestingly, Pea15 was the only of this list that is also downregulated upon LiCl+MMAE therapy (p < 0.05), while all the other proteins were brought back to control levels (*p* > 0.05). It is likely that the up or down-regulations found in these proteins are related to the impairment of Ca^2+^ signaling and/or the subsequential cascades that are affected by MMAE.

Another relevant finding was that collagens type I alpha-1 (Col1a1) and alpha-2 (Col1a2) and collagen type XI alpha-1 (Col11a1) were all substantially increased in MMAE-treated DRG (*p* < 0.05). These increased levels are in agreement with several other up-regulated proteins that are linked to inflammatory processes, such as Coagulation factor XIIIa (F13a1), Fibroblast growth factor-inducible protein 13 (Ppm1g), Prolargin (Prelp) and Parathymosin (Ptms) (*p* < 0.05). All these proteins were maintained at control levels by lithium pretreatment (*p* > 0.05 in comparison to saline).

Furthermore, Protein disulfide-isomerase A6 (Pdia6) was found to be decreased in MMAE-treated DRG, and lithium could prevent this effect (*p* < 0.05). This is an intriguing finding, since Pdia6 plays an important role in nerve damage repair [30]. Other proteins that were previously associated with nerve damage repair, including Catenin alpha-1 (Ctnna1), Inter-alpha-trypsin inhibitor heavy chain H1 (Itih1) and Coiled-coil and C2 domain containing 1B (Cc2d1b), are up-regulated in the group treated only with MMAE (*p* < 0.05) [33–35]. Note also that N-myc downstream-regulated gene 1 protein (Ndrg1), which has been associated with myelin recovery and Decorin (Dcn), previously linked to axon regeneration, are increased in MMAE-treated DRG (*p* < 0.05) [36–38]. One possible explanation for the increase in these protein levels is their action as repair mechanisms, once the nerve damage is present in MMAE. Lithium pretreatment was able to prevent the alterations of all these proteins (*p* > 0.05 in comparison to saline).

Unlike paclitaxel treatment, MMAE did not alter calpain (Capn1, Capn2), myristoylated alanine-rich C-kinase substrate (Marcks), or protein kinase C alpha (PKCα/Prkca) levels (*p* > 0.05) (Fig. 5, last panel) [10].

Together, these findings compellingly suggest that MMAE treatment is capable of causing alterations in protein levels in DRG, especially those proteins involved in intracellular Ca^2+^ signaling pathways and neuronal damage, and that LiCl co-therapy was able to prevent these changes.

### Lithium treatment does not weaken the antitumor effect of MMAE

**Fig. 6:**
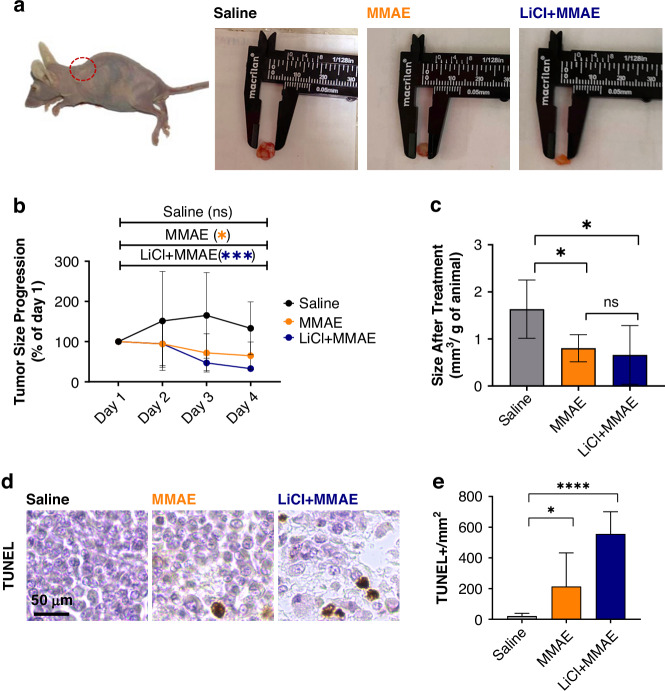
The antitumor activity of MMAE-based chemotherapy is not weakened by pretreatment with lithium. **a** Representative images of xenograft tumor of MDA-MB-231 cells induced in Balb/c nude mice, showing reduction in both MMAE and LiCl+MMAE groups in comparison to the control. **b** Time course of tumor size during the indicated treatments, showing that lithium does not harm the antitumoral activity of MMAE. **c** Size of excised tumors after indicated treatments. MMAE and LiCl+MMAE (*n* = 7 each group) had a reduction in tumor, with size final comparable to the saline (*n* = 5) treated animals (*p* < 0.01). **d** Representative image of tumors stained with TUNEL, showing presence of apoptosis in tumors from MMAE and LiCl+MMAE treated animals. **e** Graphic shows number of TUNEL positive cells per mm^2^. MMAE and LiCl+MMAE groups presented a significantly higher number of stained cells compared to the control group (*p* < 0.01 and *p* < 0.0001, respectively; *n* = 10–15 tissue sections each group) indicating cellular death in tumors treated with chemotherapy.

It is crucial to ensure that the pretreatment with lithium does not interfere in the antitumor effect of MMAE. Therefore, an in vivo experiment was performed to induce xenograft tumors, and then to measure and analyze the differences in tumor size after treatment among the groups (Fig. 6). The group treated only with MMAE and the group pretreated with lithium before MMAE administration presented shrinkage of the xenograft tumor during the experiment (Fig. 6b) and the tumor size of these groups after finishing the treatment (0.8 ± 0.3 mm^3^/g and 0.7 ± 0.7 mm^3^, respectively) was significantly smaller than the control group (1.6 ± 0.6 mm^3^; *p* < 0.05) (Fig. 6c). DAPI and mkate2 fluorescence, together with Ki-67 staining, were used to confirm the injected cells were present and proliferating in the tumor (Supplementary Fig. 4c). PET-CT images showed that lithium did not weaken the ability of MMAE to diminish the final tumor size (Supplementary Fig. b). With this PET-CT technique, nearby structures (scapula, ribs) also presented radiopharmaceutical affinity. However, we were able to at least confirm the tumor placement. TUNEL analysis demonstrated the presence of apoptosis in tumors of both MMAE and LiCl+MMAE groups, showing a significantly higher number of positive cells in animals pretreated with lithium (Fig. 6d, e). Together, these results indicate that lithium administration does not harm the antitumor effect of MMAE treatment.

## Discussion

In this study we examined the effects of MMAE, the drug cargo of several ADCs, a class of chemotherapy agents developed to better target cancer cells and to avoid off-target effects. Unfortunately, like all chemotherapy agents developed to date, there are therapy-induced side effects, notably neuropathy, with MMAE containing ADCs [39]. Previous reports of the effects of MMAE have focused on CIPN [39], which we have extended here to show that both the sensory and cognitive effects of MMAE occur. These results show that the neurological side effects can be mimicked by the drug cargo alone. This outcome is similar to the outcomes after the administration of the taxanes or the vinca alkaloids, also microtubule-based drugs like MMAE. Again, comparable to previous findings [10, 11, 40, 41], we found that lithium pretreatment before MMAE administration prevents both CIPN and CICI. The results of this study show lithium pretreatment emerges as a promising intervention in preventing both central and peripheral neuropathies induced by ADCs containing microtubule-based cargo.

Both paclitaxel and vincristine, two widely used chemotherapy agents, bind to tubulin, despite having opposite effects on microtubule stability [42–44]. It has been proposed that these drugs bind to NCS1, which leads to activation of the protease calpain, cleaving multiple proteins and ultimately decreases intracellular Ca^2+^ levels [11, 45, 46]. However, in the study reported here, the NCS1 mRNA levels were not altered by MMAE treatment in the brain. Nonetheless, the amplitude of intracellular Ca^2+^ signaling was reduced, similar to other microtubule-based agents. These results show that reduction in intracellular Ca^2+^ level is a common pathway for chemotherapy-induced neuropathy, even though NCS1 role may differ.

Proposed mechanisms of chemotherapy induced neuropathy include alteration of intracellular Ca^2+^ signaling, mitochondrial malfunction and neuroinflammation. Through a proteomic assay, we confirmed the involvement of proteins of all these pathways in our model of CIPN. Other proteins that have been implicated in the pathogenesis of taxane and vinca alkaloid induced neuropathy include protein kinase C (PKC) [10, 47, 48], interleukin 6 (IL6) [49, 50], and the components of the Wnt signaling pathway [51]. Some of the proteins (Calpain, PKC) were found to be comparable to the control group, suggesting a different pathological mechanism.

In this study we found that treatment with MMAE reduced Ca^2+^ signal amplitude and increased intracellular Ca^2+^ oscillations. This finding is comparable to reports that neuroblastoma cells (SHSY-5Y cells) or DRG isolated from mice treated with either paclitaxel or vincristine had reduced intracellular Ca^2+^ signals [52] and increased Ca^2+^ oscillations [45]. We show in this report that lithium is able to stabilize the Ca^2+^ signaling in DRG of MMAE treated mice by mobilization of intracellular Ca^2+^ compartments rather than through the influx of Ca^2+^ across the plasma membrane, as shown in the lack of response in experiments using carbachol and KCl as stimuli.

Once neuropathy is evident, the strategies to treat the condition are limited. In most cases the duration or dosage for treatment is reduced, limiting potential eradication of the tumor. Thus, there is a shift to a search for ways to prevent CIPN, including cooling of the extremities and exercise. Although there are some reports of good outcomes, they are limited and none of these strategies have been proven effective in preventing CICI [4]. Lithium pretreatment prevents intracellular Ca²⁺ reduction and functional impairments in animal models treated with taxanes [10, 11], vincristine [53], and MMAE (this report), preserving neuronal function despite chemotherapy exposure. While some reports suggest lithium may prevent neuropathy in humans [54, 55], clinical studies remain inconclusive, often insufficient by low dosages. Lithium’s modulation of NCS1 function is dose-dependent [56], and our study confirms that in MMAE-induced neuropathy, lithium dosage is critical. Dose-response curves indicate differing lithium doses are needed to prevent allodynia and loss of nociception, with half the proposed dosage preventing nociception loss but not allodynia.

Any interventions proposed to prevent CIPN and CICI must show that the ability to treat cancer is maintained. For all microtubule-based chemotherapy agents tested, specifically paclitaxel and MMAE, pretreatment with lithium did not alter the ability to shrink tumors ([11] and Figs. 4 and 5). There is evidence that lithium itself can have antitumor effects, by enhancing immune responses against the tumor, inhibiting proliferation and migration or induction of programmed cell death [57]. Our data also shows that lithium can increase apoptosis in tumoral tissues, as observed in the TUNEL assay, although the tumor size was not significantly changed by addition of lithium to the MMAE treatment. This could be due to different protocols in terms of dosage and duration of treatment.

The ultimate goal of this study was to develop a model to expedite the search for safe therapeutic compounds that will alleviate the onset and severity of neuropathy without serious side effects. Such a model now exists and an initial strategy for prevention using lithium pretreatment has been identified. While animal models provide valuable information, these findings are limited due to differences between species, highlighting the need for clinical trials in human subjects.

## Supplementary information

Supplemental Figure 1

Supplementary Figure 2

Supplementary Figure 3

Supplementary Figure 4

Supplementary Table 1

Supplementary Figures and Table Legends

## Data Availability

The datasets generated during and/or analyzed during the current study are available in the figshare repository, 10.6084/m9.figshare.26895724.v1.

## References

[1] Seretny M, Currie GL, Sena ES, Ramnarine S, Grant R, Macleod MR, et al. Incidence, prevalence, and predictors of chemotherapy-induced peripheral neuropathy: a systematic review and meta-analysis. Pain. 2014;155:2461–70. 10.1016/j.pain.2014.09.020 25261162

[2] Miller KD, Nogueira L, Devasia T, Mariotto AB, Yabroff KR, Jemal A, et al. Cancer treatment and survivorship statistics 2022. CA Cancer J Clin. 2022;72:409–36. 10.3322/caac.21731 35736631

[3] Selamat MH, Loh SY, Mackenzie L, Vardy J. Chemobrain experienced by breast cancer survivors: a meta-ethnography study investigating research and care implications. PLoS ONE. 2014;9:e108002. 10.1371/journal.pone.0108002 PMC4178068 25259847

[4] Ibrahim EY, Domenicano I, Nyhan K, Elfil M, Mougalian SS, Cartmel B, et al. Cognitive effects and depression associated with taxane-based chemotherapy in breast cancer survivors: a meta-analysis. Front Oncol. 2021;11:642382. 10.3389/fonc.2021.642382 PMC8121254 33996556

[5] Tang L, Sun C, Liu W, Wu H, Ding C. A pharmacovigilance study on antibody-drug conjugate (ADC)-related neurotoxicity based on the FDA adverse event reporting system (FAERS). Front Pharmacol. 2024;15:1362484. 10.3389/fphar.2024.1362484 PMC10879374 38384285

[6] Ahles TA, Saykin AJ. Candidate mechanisms for chemotherapy-induced cognitive changes. Nat Rev Cancer. 2007;7:192–201. 10.1038/nrc2073 PMC3329763 17318212

[7] Seigers R, Fardell JE. Neurobiological basis of chemotherapy-induced cognitive impairment: a review of rodent research. Neurosci Biobehav Rev. 2011;35:729–41. 20869395 10.1016/j.neubiorev.2010.09.006

[8] Torre M, Dey A, Woods JK, Feany MB. Elevated oxidative stress and DNA damage in cortical neurons of chemotherapy patients. J Neuropathol Exp Neurol. 2021;80:705–12. 34363676 10.1093/jnen/nlab074 PMC8660580

[9] Stagg NJ, Shen BQ, Brunstein F, Li C, Kamath AV, Zhong F, et al. Peripheral neuropathy with microtubule inhibitor containing antibody drug conjugates: challenges and perspectives in translatability from nonclinical toxicology studies to the clinic. Regul Toxicol Pharmacol. 2016;82:1–13. 27773754 10.1016/j.yrtph.2016.10.012

[10] Nguyen LD, Fischer TT, Ehrlich BE. Pharmacological rescue of cognitive function in a mouse model of chemobrain. Mol Neurodegener. 2021;16:41. 34174909 10.1186/s13024-021-00463-2 PMC8235868

[11] Mo M, Erdelyi I, Szigeti-Buck K, Benbow JH, Ehrlich BE. Prevention of paclitaxel-induced peripheral neuropathy by lithium pretreatment. FASEB J. 2012;26:4696–709. 22889832 10.1096/fj.12-214643 PMC3475250

[12] Chang HP, Cheung YK, Shah DK. Whole-body pharmacokinetics and physiologically based pharmacokinetic model for monomethyl auristatin e (Mmae). J Clin Med. 2021;10:1332. 10.3390/jcm10061332 PMC8004929 33807057

[13] Yip V, Lee MV, Saad OM, Ma S, Khojasteh SC, Shen BQ. Preclinical characterization of the distribution, catabolism, and elimination of a polatuzumab vedotin-piiq (Polivy®) antibody–drug conjugate in sprague dawley rats. J Clin Med. 2021;10:1323. 10.3390/jcm10061323 PMC8004598 33806916

[14] Pellow S, Chopin P, File SE, Briley M. Validation of open: closed arm entries in an elevated plus-maze as a measure of anxiety in the rat. J Neurosci Methods. 1985;14:149–67. 2864480 10.1016/0165-0270(85)90031-7

[15] Denninger JK, Smith BM, Kirby ED. Novel object recognition and object location behavioral testing in mice on a budget. J Visual Exp. 2018;141:10–3791. 10.3791/58593 PMC6800058 30531711

[16] Amaya F, Wang H, Costigan M, Allchorne AJ, Hatcher JP, Egerton J, et al. The voltage-gated sodium channel Nav1.9 is an effector of peripheral inflammatory pain hypersensitivity. J Neurosci. 2006;26:12852–60. 17167076 10.1523/JNEUROSCI.4015-06.2006 PMC6674969

[17] Deuis JR, Dvorakova LS, Vetter I. Methods used to evaluate pain behaviors in rodents. Front Mol Neurosci. 2017;10:284. 10.3389/fnmol.2017.00284 PMC5592204 28932184

[18] Dixon WJ, Mood AM. A method for obtaining and analyzing sensitivity data. J Am Stat Assoc. 1948;43:109–26.

[19] Sleigh JN, Weir GA, Schiavo G. A simple, step-by-step dissection protocol for the rapid isolation of mouse dorsal root ganglia. BMC Res Notes. 2016;9:82. 26864470 10.1186/s13104-016-1915-8 PMC4750296

[20] Adur J, Carvalho HF, Cesar CL, Casco VH. Nonlinear microscopy techniques: principles and biomedical applications. Microsc Anal. 2016:121–49.

[21] Fonseca MC, França A, Florentino RM, Fonseca RC, Lima Filho ACM, Vidigal PTV, et al. Cholesterol-enriched membrane microdomains are needed for insulin signaling and proliferation in hepatic cells. Am J Physiol Gastrointest Liver Physiol. 2018;315:80. 10.1152/ajpgi.00008.2018 PMC6109708 29471671

[22] Guerra MT, Florentino RM, Franca A, Lima Filho AC, Dos Santos ML, Fonseca RC, et al. Expression of the type 3 InsP3 receptor is a final common event in the development of hepatocellular carcinoma. Gut. 2019;68:1676–87. 31315892 10.1136/gutjnl-2018-317811 PMC7087395

[23] Rodrigues-Ribeiro L, Melo-Braga MN, Kjeldsen F, Gómez-Mendoza DP, Verano-Braga T. Assessment of protein extraction and digestion efficiency of well-established shotgun protocols for heart proteomics. Anal Biochem. 2019;578:51–59. 31085165 10.1016/j.ab.2019.05.006

[24] Guimarães E, MacHado R, Fonseca MC, França A, Carvalho C, Araújo E Silva AC, et al. Inositol 1, 4, 5-trisphosphate-dependent nuclear calcium signals regulate angiogenesis and cell motility in triple negative breast cancer. PLoS ONE. 2017;12:0175041. 10.1371/journal.pone.0175041 PMC5380351 28376104

[25] Van Acker N, Ragé M, Sluydts E, Knaapen MW, De Bie M, Timmers M, et al. Automated PGP9.5 immunofluorescence staining: a valuable tool in the assessment of small fiber neuropathy? BMC Res Notes. 2016;9:280. 27215701 10.1186/s13104-016-2085-4 PMC4878004

[26] Hlubocky A, Wellik K, Ross MA, Smith BE, Hoffman-Snyder C, Demaerschalk BM, et al. Skin biopsy for diagnosis of small fiber neuropathy: a critically appraised topic. Neurologist. 2010;16:61–63. 20065802 10.1097/NRL.0b013e3181c9c303

[27] de Almeida Schirmer BG, de Araujo MR, Silveira MB, Pereira JM, Vieira LC, Alves CG, et al. Comparison of [18F]Fluorocholine and [18F]Fluordesoxyglucose for assessment of progression, lung metastasis detection and therapy response in murine 4T1 breast tumor model. Appl Radiat Isotopes. 2018;140:278–88. 10.1016/j.apradiso.2018.07.032 30081351

[28] Fonseca MC, Marazzi-Diniz PHS, Leite MF, Ehrlich BE. Calcium signaling in chemotherapy-induced neuropathy. Cell Calcium. 2023;113:102762. 37244172 10.1016/j.ceca.2023.102762

[29] Nguyen LD, Ehrlich BE. Cellular mechanisms and treatments for chemobrain: insight from aging and neurodegenerative diseases. EMBO Mol Med. 2020;12:12075. 10.15252/emmm.202012075 PMC7278555 32346964

[30] Dolphin AC. Voltage-gated calcium channels and their auxiliary subunits: physiology and pathophysiology and pharmacology. J Physiol. 2016;594:5369–90. 27273705 10.1113/JP272262 PMC5043047

[31] Jiménez CR, Stam FJ, Li KW, Gouwenberg Y, Hornshaw MP, De Winter F, et al. Proteomics of the injured rat sciatic nerve reveals protein expression dynamics during regeneration. Mol Cell Proteom. 2005;4:120–32. 10.1074/mcp.M400076-MCP200 15509515

[32] Luo J, Xie M, Peng C, Ma Y, Wang K, Lin G, et al. Protein disulfide isomerase A6 promotes the repair of injured nerve through interactions with spastin. Front Mol Neurosci. 2022;15:950586. 10.3389/fnmol.2022.950586 PMC9449696 36090256

[33] Shibuya Y, Yasuda H, Tomatsuri M, Mizoguchi A, Takeichi M, Shimada K, et al. αN-Catenin expression in the normal and regenerating chick sciatic nerve. J Neurocytol. 1996;25:615–24. 9013423 10.1007/BF02284828

[34] Li J, Liu G. Lentiviral Injection of Inter-α-Trypsin inhibitor heavy chain 4 promotes female spinal cord injury mice recuperation by diminishing peripheral and central inflammation. 2024:1–8. 10.1007/s10753-024-02196-y 39648260

[35] Sophie B, Jacob H, Jordan VPJS, Yungki P, Laura FM, Yannick P. YAP and TAZ regulate Cc2d1b and Purβ in schwann cells. Front Mol Neurosci. 2019;12:177. 31379499 10.3389/fnmol.2019.00177 PMC6650784

[36] King RH, Chandler D, Lopaticki S, Huang D, Blake J, Muddle JR, et al. Ndrg1 in development and maintenance of the myelin sheath. Neurobiol Dis. 2011;42:368–80. 21303696 10.1016/j.nbd.2011.01.030

[37] Lemons ML, Barua S, Abanto ML, Halfter W, Condic ML. Adaptation of sensory neurons to hyalectin and decorin proteoglycans. J Neurosci. 2005;25:4964–73. 15901777 10.1523/JNEUROSCI.0773-05.2005 PMC6724852

[38] Staff NP, Grisold A, Grisold W, Windebank AJ. Chemotherapy-induced peripheral neuropathy: a current review. Ann Neurol. 2017;81:772–81. 28486769 10.1002/ana.24951 PMC5656281

[39] Fu Z, Gao C, Wu T, Wang L, Li S, Zhang Y, et al. Peripheral neuropathy associated with monomethyl auristatin E-based antibody-drug conjugates. iScience. 2023;26:107778. 37727735 10.1016/j.isci.2023.107778 PMC10505985

[40] Pourmohammadi N, Alimoradi H, Mehr SE, Hassanzadeh G, Hadian MR, Sharifzadeh M, et al. Lithium Attenuates peripheral neuropathy induced by paclitaxel in rats. Basic Clin Pharm Toxicol. 2012;110:231–7. 10.1111/j.1742-7843.2011.00795.x 21917116

[41] Alwhaibi AM, Alshamrani AA, Alenazi MA, Altwalah SF, Alameel NN, Aljabali NN, et al. Vincristine-Induced neuropathy in patients diagnosed with solid and hematological malignancies: the role of dose rounding. J Clin Med. 2023;12:5662. 10.3390/jcm12175662 PMC10488791 37685729

[42] Muthuraman A, Singh N, Jaggi AS. Protective effect of Acorus calamus L. in rat model of vincristine induced painful neuropathy: an evidence of anti-inflammatory and anti-oxidative activity. Food Chem Toxicol. 2011;49:2557–63. 21756962 10.1016/j.fct.2011.06.069

[43] Schiff PB, Fant J, Horwitz SB. Promotion of microtubule assembly in vitro by taxol [19-667]. Nature. 1979;277:665–7. 10.1038/277665a0 423966

[44] Burgoyne RD. Neuronal calcium sensor proteins: Generating diversity in neuronal Ca 2+ signalling. Nat Rev Neurosci. 2007;8:182–93. 17311005 10.1038/nrn2093 PMC1887812

[45] Boehmerle W, Splittgerber U, Lazarus MB, McKenzie KM, Johnston DG, Austin DJ, et al. Paclitaxel induces calcium oscillations via an inositol 1,4,5-trisphosphate receptor and neuronal calcium sensor 1-dependent mechanism. Proc Natl Acad Sci USA. 2006;103:18356–61. 17114292 10.1073/pnas.0607240103 PMC1838755

[46] Blachford C, Ćelić A, Petri ET, Ehrlich BE. Discrete proteolysis of neuronal calcium sensor-1 (NCS-1) by μ-calpain disrupts calcium binding. Cell Calcium. 2009;46:257–62. 19732951 10.1016/j.ceca.2009.08.002 PMC2763996

[47] He Y, Wang ZJ. Nociceptor beta II, delta, and epsilon isoforms of PKC differentially mediate paclitaxel-induced spontaneous and evoked pain. J Neurosci. 2015;35:4614–25. 25788678 10.1523/JNEUROSCI.1580-14.2015 PMC4363388

[48] Miyano K, Tang HBin, Nakamura Y, Morioka N, Inoue A, Nakata Y. Paclitaxel and vinorelbine, evoked the release of substance P from cultured rat dorsal root ganglion cells through different PKC isoform-sensitive ion channels. Neuropharmacology. 2009;57:25–32. 19376141 10.1016/j.neuropharm.2009.04.001

[49] Chen EI, Crew KD, Trivedi M, Awad D, Maurer M, Kalinsky K, et al. Identifying predictors of taxane-induced peripheral neuropathy using mass spectrometry-based proteomics technology. PLoS ONE. 2015;10:e0145816. 26710119 10.1371/journal.pone.0145816 PMC4692419

[50] Huehnchen P, Muenzfeld H, Boehmerle W, Endres M. Blockade of IL-6 signaling prevents paclitaxel-induced neuropathy in C57Bl/6 mice. Cell Death Dis. 2020;11:45. 31969555 10.1038/s41419-020-2239-0 PMC6976596

[51] Resham K, Sharma SS. Pharmacological interventions targeting Wnt/β-catenin signaling pathway attenuate paclitaxel-induced peripheral neuropathy. Eur J Pharm. 2019;864:172714. 10.1016/j.ejphar.2019.172714 31586636

[52] Benbow JH, Mann T, Keeler C, Fan C, Hodsdon ME, Lolis E, et al. Inhibition of paclitaxel-induced decreases in calcium signaling. J Biol Chem. 2012;287:37907–16. 22988235 10.1074/jbc.M112.385070 PMC3488062

[53] Alimoradi H, Pourmohammadi N, Mehr SE, Hassanzadeh G, Hadian MR, Sharifzadeh M, et al. Effects of lithium on peripheral neuropathy induced by vincristine in rats. Acta Med Iran. 2012;50:373–9. 22837115

[54] Najafi S, Heidarali Z, Rajabi M, Omidi Z, Zayeri F, Salehi M, et al. Lithium and preventing chemotherapy-induced peripheral neuropathy in breast cancer patients: a placebo-controlled randomized clinical trial. Trials. 2021;22:835. 34819131 10.1186/s13063-021-05800-w PMC8611897

[55] Wadia RJ, Stolar M, Grens C, Ehrlich BE, Chao HH. The prevention of chemotherapy induced peripheral neuropathy by concurrent treatment with drugs used for bipolar disease: a retrospective chart analysis in human cancer patients. Oncotarget. 2018;9:7322–31. 29484113 10.18632/oncotarget.23467 PMC5800905

[56] Schlecker C, Boehmerle W, Jeromin A, DeGray B, Varshney A, Sharma Y, et al. Neuronal calcium sensor-1 enhancement of InsP3 receptor activity is inhibited by therapeutic levels of lithium. J Clin Investig. 2006;116:1668–74. 16691292 10.1172/JCI22466 PMC1459068

[57] Ma J, Tang L, Tan Y, Xiao J, Wei K, Zhang X, et al. Lithium carbonate revitalizes tumor-reactive CD8+ T cells by shunting lactic acid into mitochondria. Nat Immunol. 2024;25:552–61. 38263463 10.1038/s41590-023-01738-0 PMC10907288

