# Supplementary figures and images for: Chemotherapy-induced neuropathy in monomethyl Auristatin E treatment: prevention by lithium
Source: Br J Cancer. 2025 Jul 1;133(5):604–14. doi: 10.1038/s41416-025-03020-6 (PMC12405523; doi:10.1038/s41416-025-03020-6)

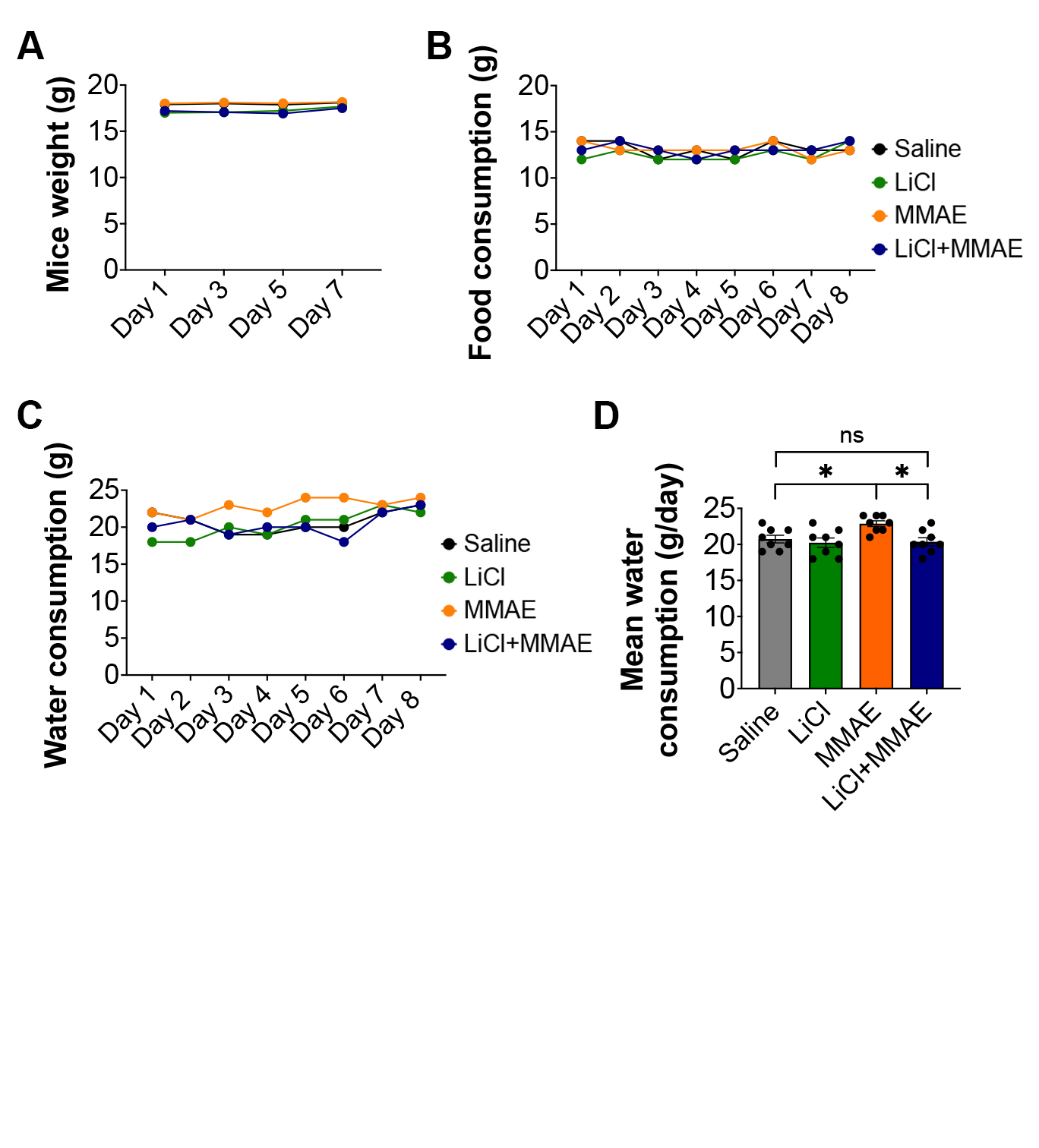

Supplement: Supplementary file 1 — Supplemental Figure 1 [file 41416_2025_3020_MOESM1_ESM.tif]

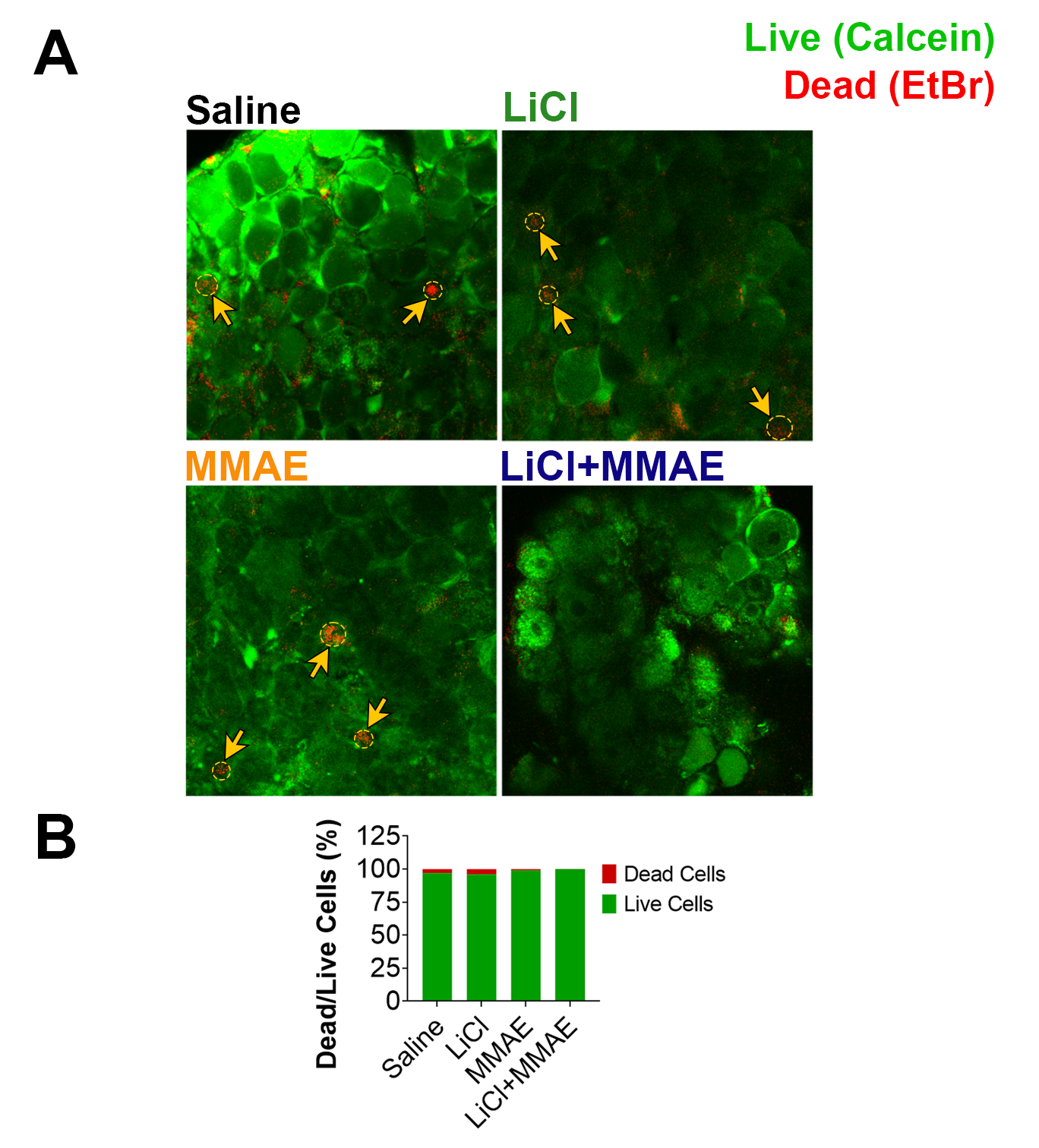

Supplement: Supplementary file 2 — Supplementary Figure 2 [file 41416_2025_3020_MOESM2_ESM.tif]

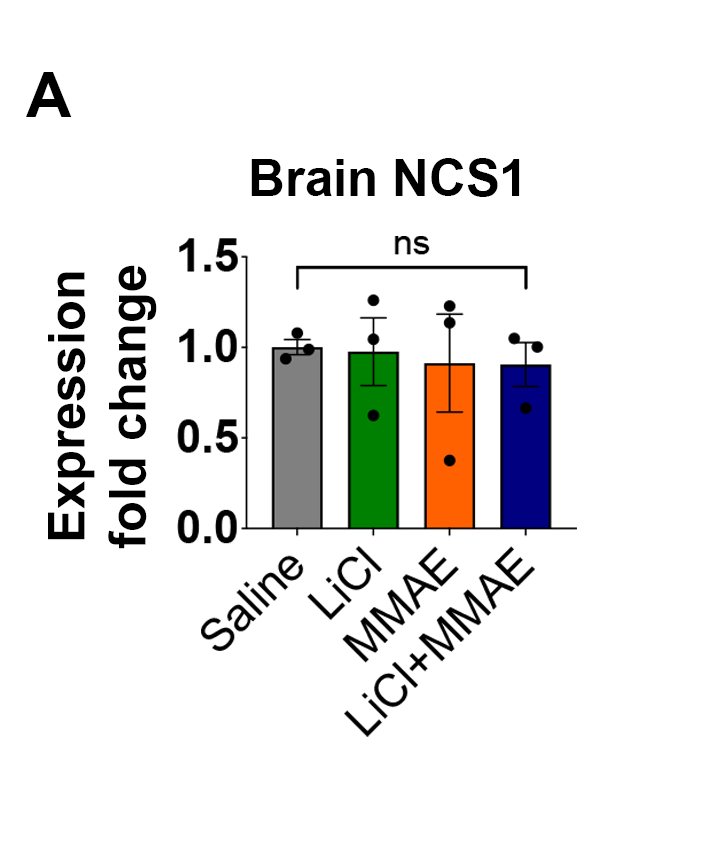

Supplement: Supplementary file 3 — Supplementary Figure 3 [file 41416_2025_3020_MOESM3_ESM.tif]

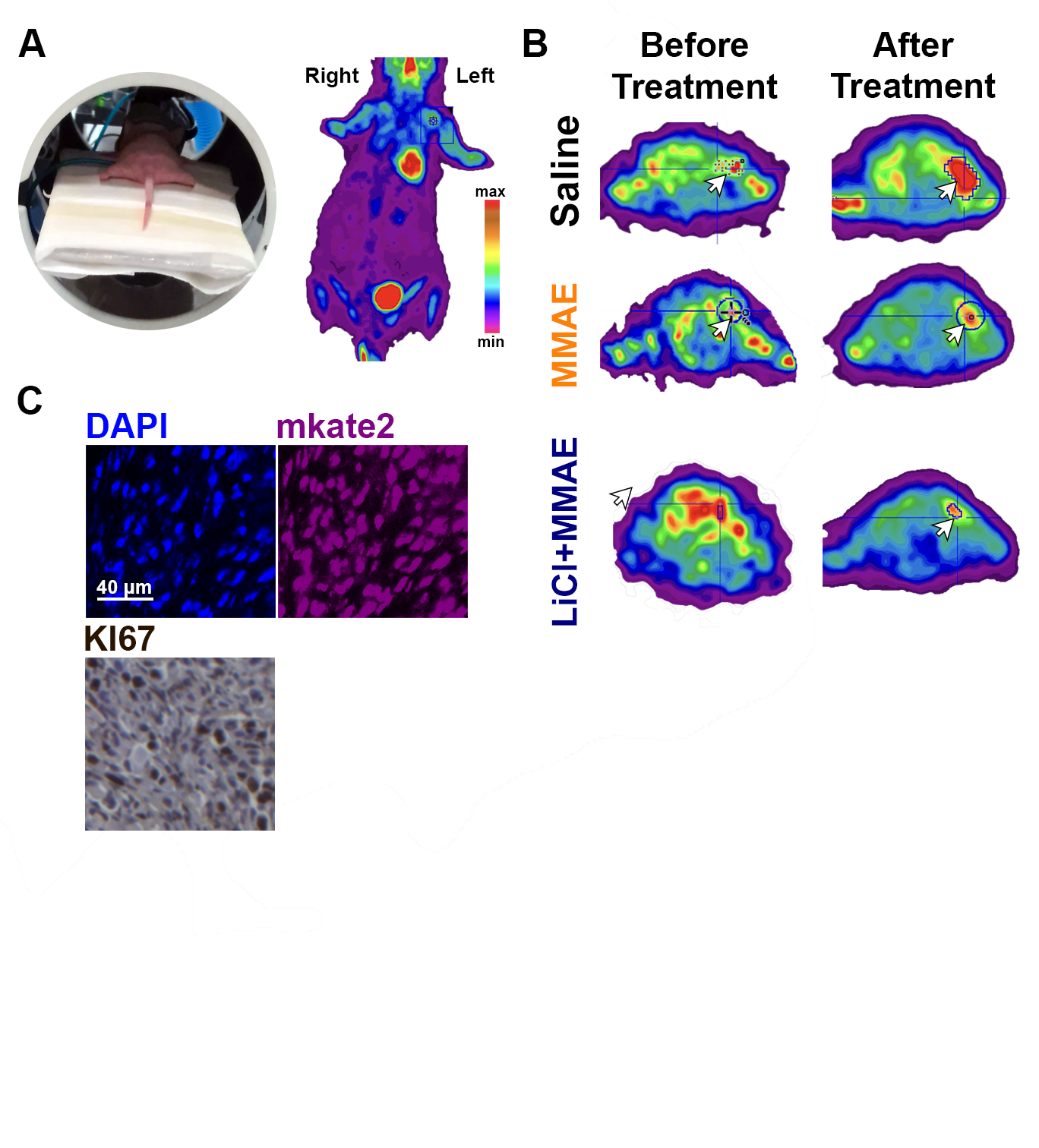

Supplement: Supplementary file 4 — Supplementary Figure 4 [file 41416_2025_3020_MOESM4_ESM.tif]
