# Supplementary material for: Chemotherapy-induced neuropathy in monomethyl Auristatin E treatment: prevention by lithium
Source: Br J Cancer. 2025 Jul 1;133(5):604–14. doi: 10.1038/s41416-025-03020-6 (PMC12405523; doi:10.1038/s41416-025-03020-6)
Supplement: Supplementary file 6 — Supplementary Figures and Table Legends [file 41416_2025_3020_MOESM6_ESM.docx]

**Supplementary Table 1.** Proteomic data of DRG comparing LiCl, MMAE and LiCl+MMAE groups to saline. Values of p are listed to ensure significance of the alterations.

**Supplementary Figure 1.** A) Mice weight and B) food consumption are not altered by treatments. C) and D) Water consumption is slightly increased in MMAE treated animals.

**Supplementary Figure 2.** A) Representative images of the Live and Dead assay in DRG treated with MMAE and LiCl show generally the same patterns in terms of cell death. B) Quantification shows a low and generalized number of dead cells in DRG tissues.

**Supplementary Figure 3.** A) RT-PCR of brain NCS1 shows no significant difference in expression among the groups.

**Supplementary Figure 4.** A) Representative images of the apparatus used for the PET measurements and mouse whole-body imaging. B) Tumor visualization before and after the treatment with MMAE, LiCl+MMAE or saline. White arrows indicate the tumoral area. C) Representative images of MDA-MB-231 xenograft tumor excised from control animals. In blue, DAPI fluorescence shows nuclei. In far-red (magenta), mkate2 staining demonstrates the presence of MDA-MB-231 cells (transfected to present fluorescence). In brown, Ki-67 staining shows proliferation in the tumor cells. Scale bar 40 μm.
